# Bringing action into the picture. How action influences visual awareness

**DOI:** 10.3758/s13414-019-01781-w

**Published:** 2019-06-05

**Authors:** Anna Anzulewicz, Justyna Hobot, Marta Siedlecka, Michał Wierzchoń

**Affiliations:** Consciousness Lab, Institute of Psychology, Jagiellonian University, Ingardena 6, 30-060 Kraków, Poland

**Keywords:** Visual awareness, Perception and Action, Consciousness

## Abstract

This article discusses how the analysis of interactions between action and awareness allows us to better understand the mechanisms of visual awareness. We argue that action is one of several factors that influence visual awareness and we provide a number of examples. We also discuss the possible mechanisms that underlie these influences on both the cognitive and the neural levels. We propose that action affects visual awareness for the following reasons: (1) it serves as additional information in the process of evidence accumulation; (2) it restricts the number of alternatives in the decisional process; (3) it enables error detection and performance monitoring; and (4) it triggers attentional mechanisms that modify stimulus perception. We also discuss the possible neuronal mechanisms of the aforementioned effects, including feedback-dependent prefrontal cortex modulation of the activity of visual areas, error-based modulation, interhemispheric inhibition of motor cortices, and attentional modulation of visual cortex activity triggered by motor processing.

## What factors influence awareness

What factors shape visual awareness? This question addresses the central problem in this research area: the cognitive and neural mechanisms of awareness. Multiple theories offer different approaches to the problem (Block, 2011; Dehaene, 2008; Lamme, 2010; Lau & Rosenthal, 2011, to list just a few), so we start by explicitly stating the one assumed in this paper. Following the hierarchical view (Lau & Rosenthal, 2011), we do not distinguish visual awareness from conscious access, assuming that the former requires the latter. In this approach, visual awareness can be measured by different subjective scales that allow assessment of changes in visual awareness level, such as the Perceptual Awareness Scale or Confidence Ratings (Norman & Price, 2015; Sandberg et al., 2010). The cognitive mechanism of visual awareness defined as such is not clear.

There has been extensive research on the factors that influence visual awareness at the neural, behavioral, and subjective levels. One of the contexts in which these factors have been studied pertains to the problem of the gradualness of visual awareness, which starts with the question of whether visual awareness is an all-or-none or gradual phenomenon; that is, whether there are intermediate states between complete unawareness and full awareness of perceptual content. This has been widely debated on the theoretical level (Bayne, Hohwy, & Owen, 2016; Jonkisz, Wierzchoń, & Binder, 2017), but the neural and cognitive mechanisms remain undetermined (Dehaene, Sergent, & Changeux, 2003; Fazekas & Overgaard, 2018; Kouider et al., 2010). The notion of the gradualness of visual awareness (i.e., degrees of conscious content, see Bayne, Howhy, & Owen, 2016) can be understood in at least two ways: First, it can pertain to the dynamics of the transition from unconscious to conscious processing, or accumulation of perceptual evidence in time (Anzulewicz et al., 2015, but also see Dehaene, 2008), as reflected in the question “is there a clear threshold that a representation needs to pass to enable conscious access?” Second, it could be related to the quality of conscious content, namely how many levels of stimulus clarity can be distinguished between complete unawareness and full awareness of a stimulus (Ramsoy & Overgaard, 2004; see the phenomenal dimension of graded consciousness – Jonkisz, Wierzchoń, & Binder, 2017). For the sake of clarity, in this paper we focus on the latter approach. The evidence available so far demonstrates that, on a subjective level, awareness is typically graded, as evidenced by the variability in visual awareness ratings (Anzulewicz et al. 2015; Windey, Gevers, & Cleeremans, 2013). To put it simply, when subjective awareness measures such as the Perceptual Awareness Scale (PAS) are applied in visual tasks, participants tend to use all available scale points, suggesting the gradualness of visual awareness (Overgaard et al., 2006). Interestingly, the ratings pattern can be more or less graded, depending on additional factors. It has been proposed that gradualness is influenced by the level of information processing imposed by a given task (Kouider et al. 2010; Windey, Gevers, & Cleeremans, 2013) and by the involvement of attentional processing, for example, attentional amplification or attentional tuning (Fazekas & Overgaard, 2018). The latter view states that attention influences visual stimulus representation at different levels and therefore changes the quality of this representation (its intensity, distinctiveness, and stability) and thus visual awareness. It has been recently pointed out that the set of factors that influence gradualness of visual awareness should be better specified and extended (Anzulewicz & Wierzchoń, 2018). First, even if attentional mechanisms are the most important factor influencing awareness (Kveraga, Ghuman, & Bar, 2007; Pinto, van Gaal, de Lange, Lamme, & Seth, 2015), their influence might differ at different levels of stimulus processing (Anzulewicz & Wierzchoń, 2018). Second, other factors may influence visual awareness, and their role has not yet been investigated. Third, it seems possible that the aforementioned factors influence not only the gradualness of visual awareness but also other aspects of visual awareness (e.g., awareness threshold, awareness level, etc.).

The majority of studies that investigate the factors that influence visual awareness point to attention as the main factor (Fuller, Park, & Carrasco, 2009; Zhang & Luck, 2009). However, some other factors have recently been proposed, such as expectations (Kok, Jehee, & de Lange, 2012; Melloni, Schwiedrzik, Muller, Rodriguez, & Singer, 2011; Pinto, van Gaal, de Lange, Lamme, & Seth, 2015), previous experience (Cleeremans & French, 2002), context (Kveraga, Ghuman, & Bar, 2007), and performance feedback (Siedlecka, Wereszczyński, Paulewicz, & Wierzchoń, 2019). This empirical evidence is accompanied by the development of theoretical models of visual awareness that also discuss different factors that may influence visual awareness (Fazekas & Overgaard, 2018; Hohwy, 2013; Park & Tallon-Baudry, 2014). Interestingly, none of the theories have proposed action as a relevant factor.

Here, we present an analysis of the ways in which action may affect visual awareness. It has been demonstrated that the action–perception loop plays a vital role in what is perceived by an individual (Donnarumma et al., 2017; Hecht et al., 2001). Nevertheless, the most influential theories of visual awareness do not consider the role of action. On the contrary, most of the theories propose that visual awareness is based solely on perceptual evidence (e.g., Block, 2011; Dehaene, Sergent & Changeux, 2003; Lamme, 2010, and many more). In this paper, we bring action into the picture and investigate how it can influence visual awareness.

## Empirical evidence for the influence of action on visual awareness

It has been demonstrated that action and perception are closely coupled (Donnarumma et al., 2017; Hecht et al., 2001). Interestingly, however, little is known about how motor-related information influences visual awareness.

Over recent years, there has been a growing amount of empirical evidence showing the relation between action and visual awareness. This issue has usually been studied with a perceptual discrimination task (e.g., orientation discrimination, shape discrimination, etc.), in addition to which participants were asked to rate their stimulus awareness using a subjective scale (e.g., Perceptual Awareness Scale (PAS) – Ramsøy & Overgaard, 2004, or confidence ratings (CR) – Cheesman & Merikle, 1986). It has been shown that discrimination response affects the level of visual awareness. For instance, Fleming and colleagues (Fleming, Maniscalco, Ko, Amendi, Ro, & Lau, 2015) applied unilateral single-pulse transcranial magnetic stimulation (TMS) to the dorsal premotor cortex associated with either a chosen or an unchosen response, either before or immediately after participants provided a discrimination response. The results showed that confidence decreased when TMS to the premotor cortex was incongruent with participants’ correct response, suggesting that motor-related information contributes to visual awareness (as measured with CR). In another study, participants, prior to providing a PAS rating, responded to a cue presented on the screen (Siedlecka, Hobot, Skóra, Paulewicz, Timmermans, & Wierzchoń, 2018). The cue-elicited response was neutral, congruent, or incongruent with the correct response to the stimulus given after PAS rating. Participants reported a higher level of stimulus awareness in both the congruent and the incongruent conditions compared to the neutral one. This result suggests that motor response, no matter whether congruent or incongruent but overlapping with stimulus-related response, increases the level of visual awareness. Similarly, it has been shown that awareness level was higher in trials in which preparatory motor activity was present, irrespective of whether it was congruent with the correct response to the stimulus (Gajdos, Fleming, Saez Garcia, Weindel, & Davranche, 2018). Moreover, carrying out or even preparing a motor response might provide information about its accuracy. In our recent study we observed that perceptual awareness ratings were sensitive to the outcomes of performance monitoring (Siedlecka, Wereszczyński, Paulewicz, & Wierzchoń, 2019). Participants reported lower visual awareness in a given trial if their previous perceptual discrimination response had been incorrect. We observed this effect in conditions with and without explicit accuracy feedback, but it was stronger when feedback was given.

So far, a few non-mutually exclusive explanations of the effects mentioned above have been suggested, including decision-making and confidence models (Fleming et al., 2015), performance monitoring (Boldt & Yeung, 2015; Gajdos et al., 2018; Siedlecka et al., 2018), second-order models of metacognition (Fleming & Daw, 2017), and higher-order theories of consciousness (Siedlecka et al., 2018). However, none of these explanations seem satisfactory as they do not advocate for a mechanism explaining how action may influence visual awareness.

## How action may influence visual awareness

How may action and other non-perceptual types of information affect visual awareness? On the one hand, it may be expected that the more evidence that is present, the more aware of a stimulus we are, thus an increase in awareness is reported. In other words, motor response (e.g., in the perceptual discrimination task) provides additional evidence that adds to the evidence in the accumulation process, thus resulting in visual awareness change. Alternatively, action associated with a given stimulus representation may restrict the influence of the alternative representation in the evidence accumulation process (e.g., when a participant responds “left” in the orientation discrimination task, the evidence for the “right” stimulus may be overridden). Finally, an erroneous motor response may lead to error detection and in consequence change in reported awareness.

In all the aforementioned cases, visual awareness may vary in different ways. We may observe not only increased or decreased awareness ratings, but also changes in metacognitive access, measured as the correlation between discrimination task accuracy and visual awareness ratings. This correlation might be independent of visibility itself (correlation is high both when we know we saw something accurately and when we know we did not see accurately; the latter case pertains to the accuracy of metacognitive judgments rather than visual awareness itself).

What are the neural mechanisms of the effects of action on visual awareness? One possible mechanism is suggested by results of studies investigating the relation between partial muscular activations and the level of perceptual confidence. Gajdos et al. (2018) observed higher perceptual confidence in trials with motor preparatory activity (no matter whether it was ipsilateral or contralateral to the provided response) as measured with electromyographic (EMG) partial muscular activations. Thus, the results of this study provide conceptual support for the interpretation that the presence of additional, motor-related evidence influences visual awareness (note that the evidence is not direct as it was a correlational study).

The integration of action evidence into visual awareness ratings may be related to the prefrontal cortex (PFC) modulatory processing, which enables additional evidence accumulation. However, this additional evidence does not significantly affect discrimination task performance, which suggests that it is added as evidence at a later stage of visual processing. Importantly, it seems probable that participants are not aware of the EMG pre-activations, thus suggesting that the content of the additional evidence is not conscious and possibly not processed in the PFC.

In detection and identification tasks, competitive evidence may be accumulated for response initiation or selection. In the former case, evidence for facilitation and for inhibition of response compete with each other; the latter may involve alternative motor evidence. Regardless of whether the actions are executed with the same hand or with two hands, facilitation and inhibition mechanisms (interhemispheric and/or contralateral) are involved in this case as well. The information for response selection may be maintained and then fed to visual awareness ratings. This requires the engagement of response-selection mechanisms that are guided by prefrontal and posterior cortical circuits (Mostofsky & Simmonds, 2008). While the parietal cortex is involved in activating possible stimulus-response associations (Bunge, Hazeltine, Scanlon, Rosen, & Gabrieli, 2002), the prefrontal cortex is mainly engaged in response selection (Rowe, Toni, Josephs, Frackowiak, & Passingham, 2000).

Importantly, errors in detection and identification tasks are often followed by error-related negativity (ERN; Scheffers, Coles, Bernstein, Gehring, & Donchin, 1996; Vidal, Hasbroucq, Grapperon, & Bonnet, 2000; Meckler, Carbonnell, Ramdani, Hasbroucq, & Vidal, 2017); this is an event-related potential (ERP) that is believed to be generated in the supplementary motor area (SMA) or pre-SMA (Bonini, Burle, Liégeois-Chauvel, Régis, Chauvel, & Vidal, 2014), both of which are in turn controlled by the PFC during motor planning and action execution. The PFC could thus integrate evidence from the visual system and use action activation as additional evidence for visual awareness. There are two possible types of evidence here: positive when action refers to the expected and congruent response, but negative after an error. Thus, evidence accumulation includes the error-monitoring process; error detection (either conscious or unconscious) could be associated with a change in visual awareness.

We argue that motor evidence needs to be integrated into the evidence accumulation process in order to influence visual awareness. Since the PFC not only integrates and coordinates information from other associative areas but also controls movement and action planning (Kim & Lee, 2012; Narayanan & Laubach, 2006; Vertes, 2006), we assume it plays a major role in this process. There is evidence supporting reciprocal connections between the PFC and motor cortices (Bedwell et al., 2014). The PFC interacts with premotor and supplementary motor areas, and in consequence also with the primary motor cortex, which co-represents elementary movements, including their direction (Georgopoulos et al., 1982). We propose that the PFC integrates additional evidence for visual awareness because its activity can be modulated following errors (Narayanan & Laubach, 2008), probably due to modulation of the anterior cingulate cortex activity (Fuster, 2001). At the same time, the PFC may modulate motor cortex excitability: for example, different types of errors may influence motor cortex excitability differently, depending on whether the error is due to successful realization of an erroneous motor plan (possibly via direct feedback from motor areas) or to unsuccessful realization of an accurate motor plan (possibly via interoception or observation of one’s own behavior).

A change in reported visual awareness related to performance monitoring may occur due to the acquisition of additional information, independent of its informational value. When a participant is forced to react fast, the involvement of the PFC is reduced and the additional evidence may not be sufficiently processed (e.g., new evidence is present, but its value has not been considered). When participants know their general accuracy, they may adjust reported visibility (e.g., perceived task difficulty could affect visual awareness rating) or modulate attentional engagement in the task due to motivational factors. The engagement of the PFC in attentional processing – and thus in selective information processing – is closely related to its role in navigating goal-directed actions. Information about errors is processed in the PFC, which in turn may influence the dorsal attentional network and modulate the excitability of the visual cortex (Fernandez-Duque, Baird, & Posner, 2000). Similar modulations of visual cortex excitability may also occur due to expectations associated with motor actions and the attentional load related to the task. Given the connections between PFC and pontine cells, it is plausible that the PFC sends information to the pons and cerebellum, but the existence of such feedback processing has not yet been confirmed.

It may be questioned whether all the information that influences visual awareness necessarily needs to be mediated via the PFC. Although functional interactions between the visual and motor systems are not yet well established and there are no direct anatomic projections from the primary visual cortex to the motor and premotor areas, there are complex indirect cortico–cortical pathways throughta which visual areas may influence motor and premotor areas for the visual guidance of movements. These cortico–cortical links are believed to be mediated via the corpus callosum (Glickstein, 2000). Posterior extrastriate visual areas have reciprocal connections with the lateral intraparietal area (LIP), frontal eye fields (FEF), and dorsolateral prefrontal cortex (DLPFC), all of which are areas that are involved in visual attention and motor planning. The LIP and the FEF play a predominant role in processes related to visual-spatial attention and eye-movement planning and are firmly interconnected (Tong, 2003). Parietal areas are also involved in localization of objects and allow individuals to act on them even if they cannot recognise them. Therefore, involvement of the frontal or temporal lobes is not necessary for the execution of visually guided arm movements (Myers, Sperry, & McCurdy, 1962). This is possible due to the existence of subcortical routes. Parietal areas of the dorsal stream project to the cerebellum through connections to the pontine nuclei (Glickstein et al., 1980), which constitutes the largest subcortical pathway that links visual and motor areas. Pontine cells receive inputs from visual cortical areas, the superior colliculus and the pretectum (Burne, Azizi, Mihailoff, & Woodward, 1981; Mower, Gibson, & Glickstein, 1979) and transfer information to the cerebellar cortex, which in turn projects to the cerebral cortex (Stein & Glickstein, 1992). The other known subcortical route leads to the motor cortex via the basal ganglia (Saint-Cyr, Ungerleider, & Desimone, 1990); however, the possibility of reciprocal interactions is uncertain, and there is not enough evidence to postulate that there are direct influences from the motor cortex to visual areas via subcortical routes. Nevertheless, if they indeed exist, the role of such a connection for visual awareness should be considered, but it seems premature to conclude on its mechanisms.

To sum up, there are numerous ways in which motor behavior influences visual awareness. First, action may serve as additional information that influences visual awareness. Second, action execution may restrict the number of alternatives in the decisional process. Third, action execution may enable performance monitoring (e.g., thanks to the detection of errors or because of increased task difficulty). Fourth, action may trigger attentional mechanisms that modify stimulus perception. The first mechanism would be based on the pre-activation of the motor cortex or co-activation of motor and visual cortices, where motor-related information (possibly based on functional synchronisation) is integrated – possibly thanks to involvement of the PFC – in visual awareness together with visual information. The second mechanism pertains to the decisional process of response selection, where action restricts the alternative options available. Here, we assume that response choice inhibits its alternative by interhemispheric inhibition between bilateral M1s, the dPM, and the contralateral M1 (Mochizuki, Huang, & Rothwell, 2004). The third mechanism seems to be related to error-detection and monitoring on the neuronal level that involves ACC processing. It is conceivable that all the mechanisms may be engaged either together with or without engagement of the PFC. Moreover, one may or may not be aware of additional evidence. Motor cortex excitability and therefore motor movements may be modulated without awareness of additional evidence and/or information from the PFC that is passed through cortico-cortical or subcortical routes. The fourth mechanism requires the engagement of the PFC and top-down attentional modulation of visual cortex excitability. Here, the action influence would be indirect: action would influence attention and this in turn would affect visual awareness.

The current paper presents the hypothetical cognitive and neuronal mechanisms of how action influences visual awareness. The detailed prediction of the proposed mechanisms still requires investigation; however, empirical evidence clearly suggests that action influences visual awareness, extending the classical view on the action-perception cycle.
